# Supplementary material for: Causal Relationship between Aging and Anorexia Nervosa: A White-Matter-Microstructure-Mediated Mendelian Randomization Analysis
Source: Biomedicines. 2024 Aug 16;12(8):1874. doi: 10.3390/biomedicines12081874 (PMC11351342; doi:10.3390/biomedicines12081874)
Supplement: Supplementary file 1 [file biomedicines-12-01874-s001.zip › Supplementary Figure/Figure S4/Singe SNP plot of MR analysis for TL to ubm-b-1963.pdf]

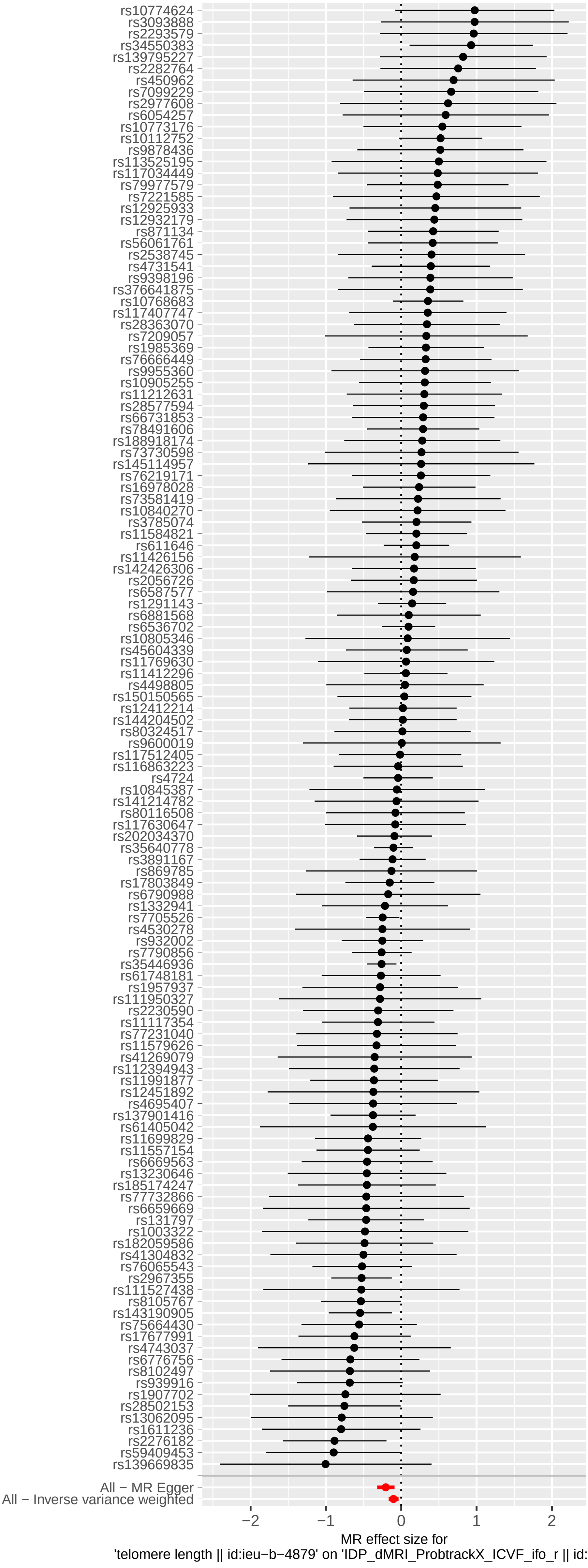
rs10774624
rs3093888
rs2293579
rs34550383
rs139795227
rs2282764
rs450962
rs7099229
rs2977608
rs6054257
rs10773176
rs10112752
rs9878436
rs113525195
rs117034449
rs79977579
rs7221585
rs12925933
rs12932179
rs871134
rs56061761
rs2538745
rs4731541
rs9398196
rs376641875
rs10768683
rs117407747
rs28363070
rs7209057
rs1985369
rs76666449
rs9955360
rs10905255
rs11212631
rs28577594
rs66731853
rs78491606
rs188918174
rs73730598
rs145114957
rs76219171
rs16978028
rs73581419
rs10840270
rs3785074
rs11584821
rs611646
rs11426156
rs142426306
rs2056726
rs6587577
rs1291143
rs6881568
rs6536702
rs10805346
rs45604339
rs11769630
rs11412296
rs4498805
rs150150565
rs12412214
rs144204502
rs80324517
rs9600019
rs117512405
rs116863223
rs4724
rs10845387
rs141214782
rs80116508
rs117630647
rs202034370
rs35640778
rs3891167
rs869785
rs17803849
rs6790988
rs1332941
rs7705526
rs4530278
rs932002
rs7790856
rs35446936
rs61748181
rs1957937
rs111950327
rs2230590
rs11117354
rs77231040
rs11579626
rs41269079
rs112394943
rs11991877
rs12451892
rs4695407
rs137901416
rs61405042
rs11699829
rs11557154
rs6669563
rs13230646
rs185174247
rs77732866
rs6659669
rs131797
rs1003322
rs182059586
rs41304832
rs76065543
rs2967355
rs111527438
rs8105767
rs143190905
rs75664430
rs17677991
rs4743037
rs6776756
rs8102497
rs939916
rs1907702
rs28502153
rs13062095
rs1611236
rs2276182
rs59409453
rs139669835
All − MR Egger
All − Inverse variance weighted
−2
−1
0
1
2
MR effect size for
'telomere length || id:ieu−b−4879' on 'IDP_dMRI_ProbtrackX_ICVF_ifo_r || id:u
